# Supplementary material for: Isl1+ Central Amygdala Neurons Coordinate Control of the Jaw and Stomach During Ingestion
Source: bioRxiv. 2026 Aug 17:2026.08.11.744231. Preprint. [Version 1] doi: 10.64898/2026.08.11.744231 (PMC13674593; doi:10.64898/2026.08.11.744231)
Supplement: Supplementary file 1 [file NIHPP2026.08.11.744231V1-supplement-1.pdf]

bioRxiv preprint doi: https://doi.org/10.64898/2026.08.11.744231; this version posted August 17, 2026. The copyright holder has placed this preprint (which was not certified by peer review) in the Public Domain. It is no longer restricted by copyright. Anyone can legally share, reuse, remix, or adapt this material for any purpose without crediting the original authors.

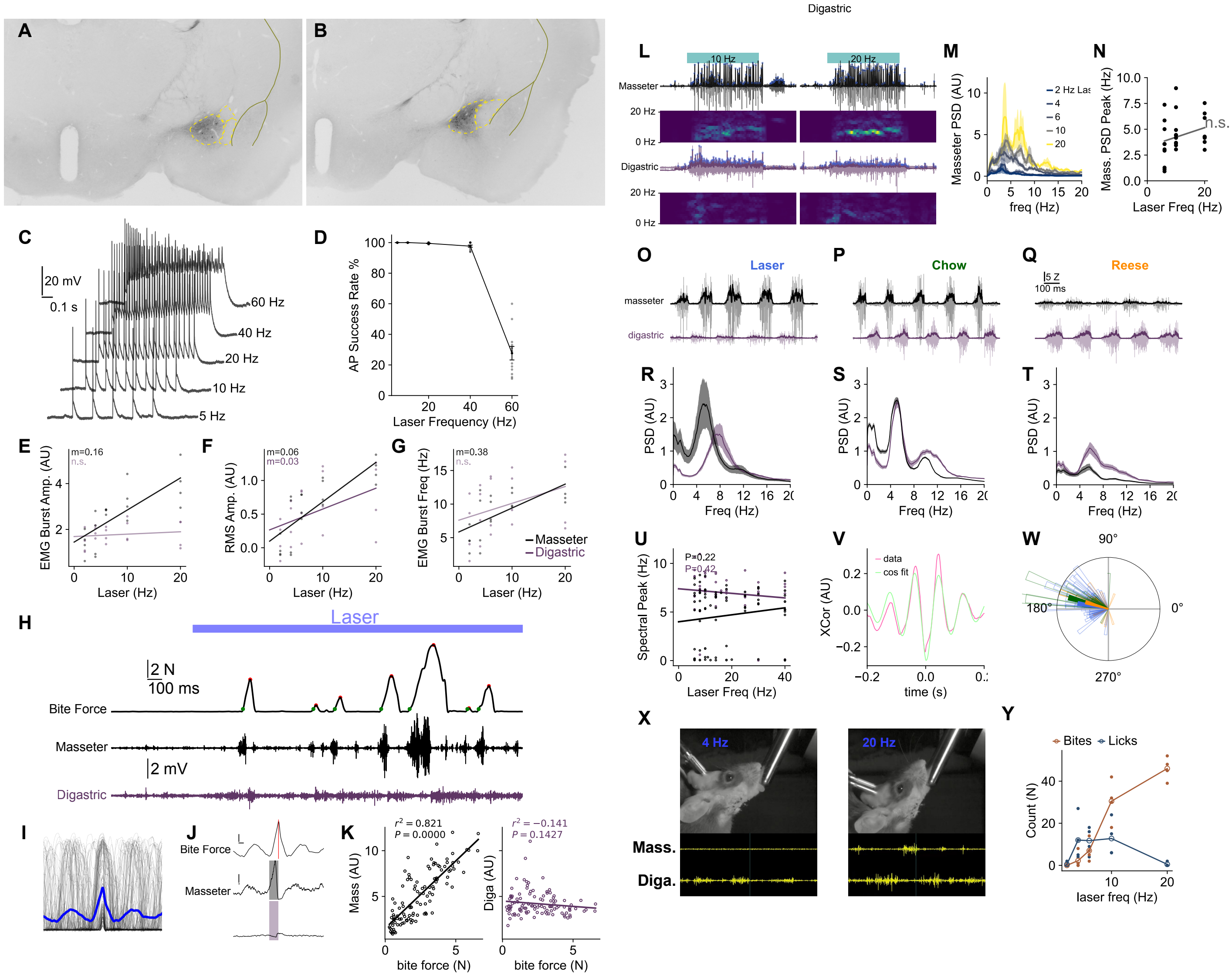

Figure S1: Stimulation of CeA^{Isl1+} neurons drives rhythmic contraction of the jaw closer masseter muscle.

(A-B) Representative images of GFP expression following injection of Cre dependent AAV into the central amygdala of $Isl1^{Cre}$ animals. Note that viral expression is restricted to the medial division of the central amygdala. (C) Representative traces from whole cell patch clamp recordings of CeA neuron expressing eYFP following injection of AAV driving Cre dependent expression of ChR2.eYFP in $Isl1^{Cre}$ mouse. Response to trains of 470 nm LED flashes. (D) Quantification of fraction of flashes within a train successfully evoking action potentials (N=12 cells, 3 mice). LED stimulation at 60 Hz lead to depolarization blockade and failure to repetitively fire. (E-G) Quantification of jaw opener (digastric) and closer (masseter) EMG signal in response to stimulation of CeA^{Isl1+} neurons. (E) Average amplitude of each burst peak is linearly related to the frequency of CeA^{Isl1+} neuron stimulation for the masseter muscle, (N=4, P=1.41e-08, slope=0.156667), but this relationship is not significant for the digastric muscle, (N=4, P=0.528). (F) In contrast, the averaged total area under the rectified EMG (RMS amplitude) is linearly related to the frequency of CeA^{Isl1+} neuron stimulation for both masseter muscle, (N=4, P=8.169-10, slope=0.062534), and for the digastric muscle, (N=4, P=0.028, slope=0.031494). (G) The average frequency of EMG bursts is linearly related to the frequency of CeA^{Isl1+} neuron stimulation for the masseter muscle, (N=4, P=2.212e-06, slope= 0.384417), but not the digastric muscle, (N=4, P=0.115). (H-K) The masseter EMG signal is linearly related to bite force. (H) Example traces of signals recorded during activation of CeA^{Isl1+} neurons. Upper trace, bite force, middle trace, masseter EMG, lower trace, digastric EMG. Blue bar above indicates initiation of laser stimulation. Red dots in the upper force trace show detected peaks in the force recording, which are used for later alignment of the EMG and force data. Green dots in the force trace indicate the time of threshold crossing for the alternate method of alignment. (I) Each peak in the force trace (red dots in A) is aligned to time zero, individual traces are overlayed in black, and the average force is plotted in blue. (J) Triggered averages of individual bites are computed for bite force (top), masseter EMG (middle), and digastric EMG (bottom). Individual bites are aligned so that the peak of recorded force is at time zero, shown by the red line. Orange patch overlaying the masseter EMG trace and blue patch overlaying the digastric EMG trace show the period of during which the EMG signal was summed for the purpose of creating the force - EMG correlation. (K) The masseter EMG activity has a tight positive correlation with measured bite force (left plot), whereas digastric EMG activity is not significantly correlated (right plot). (L-N) Rhythmic masseter EMG activity evoked by stimulation of CeA^{Isl1+} neurons has a

bioRxiv preprint doi: https://doi.org/10.64898/2026.08.11.744231; this version posted August 17, 2026. The copyright holder has placed this preprint (which was not certified by peer review) in the Public Domain. It is no longer restricted by copyright. Anyone can legally share, reuse, remix, or adapt this material for any purpose without crediting the original authors.

peak in spectral power that is insensitive to the rate of laser stimulation. (L) Example traces showing activation of the masseter (black trace top), and the digastric (purple trace) in response to a increasing frequencies of CeA^{Isl1+} neuron stimulation. Rectified integrated EMG signals (thicker lines) are used for the spectral analysis. Upper (masseter) and lower (digastric) spectrograms show the emergence of regular rhythmic activity between 4 and 10 Hz during stimulation that is marked for the masseter during stimulation of CeA^{Isl1+} neurons between 10 and 20 Hz. (M) Power spectral density estimates for masseter during stimulation of CeA^{Isl1+} cells at increasing stimulation rates. (N) No significant linear relationship exists between the predominant frequency peak of masseter spectral activity and laser stimulation rates. (O-U) Stimulation of CeA^{Isl1+} neurons releases rhythmic activity of the masseter muscle that has similar spectral characteristics as is observed in the consumption of chow. (O-Q) Representative EMG recordings during activation of CeA^{Isl1+} neurons by laser, during chow consumption, and during reese's peanut cup consumption. (R-T) Aggregate data (N=5) of masseter and digastric spectrograms corresponding to panels O,P,and Q respectively. (U) As in the previous group (Panel N), no significant different is observed between the frequency of predominant peak in spectral activity and the rate of laser stimulation, for either the masseter or digastric muscle. (V-W) The phase relationship between the masseter and digastric muscle during stimulation of CeA^{Isl1+} neurons is similar to that observed during consumption of food. V. Example data showing representative fitting of a damped cosine function to the cross-correlogram of the masseter and digastric recording. W. Radial plot of fit phase term (plot angle), and amplitude term (bar height). (X-Y) Stimulation of CeA^{Isl1+} neurons sometimes released licking behaviors. X. Example still frames from videos recorded while mouse was presented with dry sipper and the laser was activated at 4 (left frame) or 20 Hz (right frame). In this mouse, EMG from the masseter and digastric were recorded simultaneously. A lick is elicited with 4 Hz stimulation, whereas at 20 Hz the mouse is biting the sipper (note the masseter activation). (Y) Quantification of number of bites and licks evoked by different rates of laser activation ranging from 2-20 Hz, when the mouse is presented with a dry sipper.

bioRxiv preprint doi: https://doi.org/10.64898/2026.08.11.744231; this version posted August 17, 2026. The copyright holder has placed this preprint (which was not certified by peer review) in the Public Domain. It is no longer restricted by copyright. Anyone can legally share, reuse, remix, or adapt this material for any purpose without crediting the original authors.

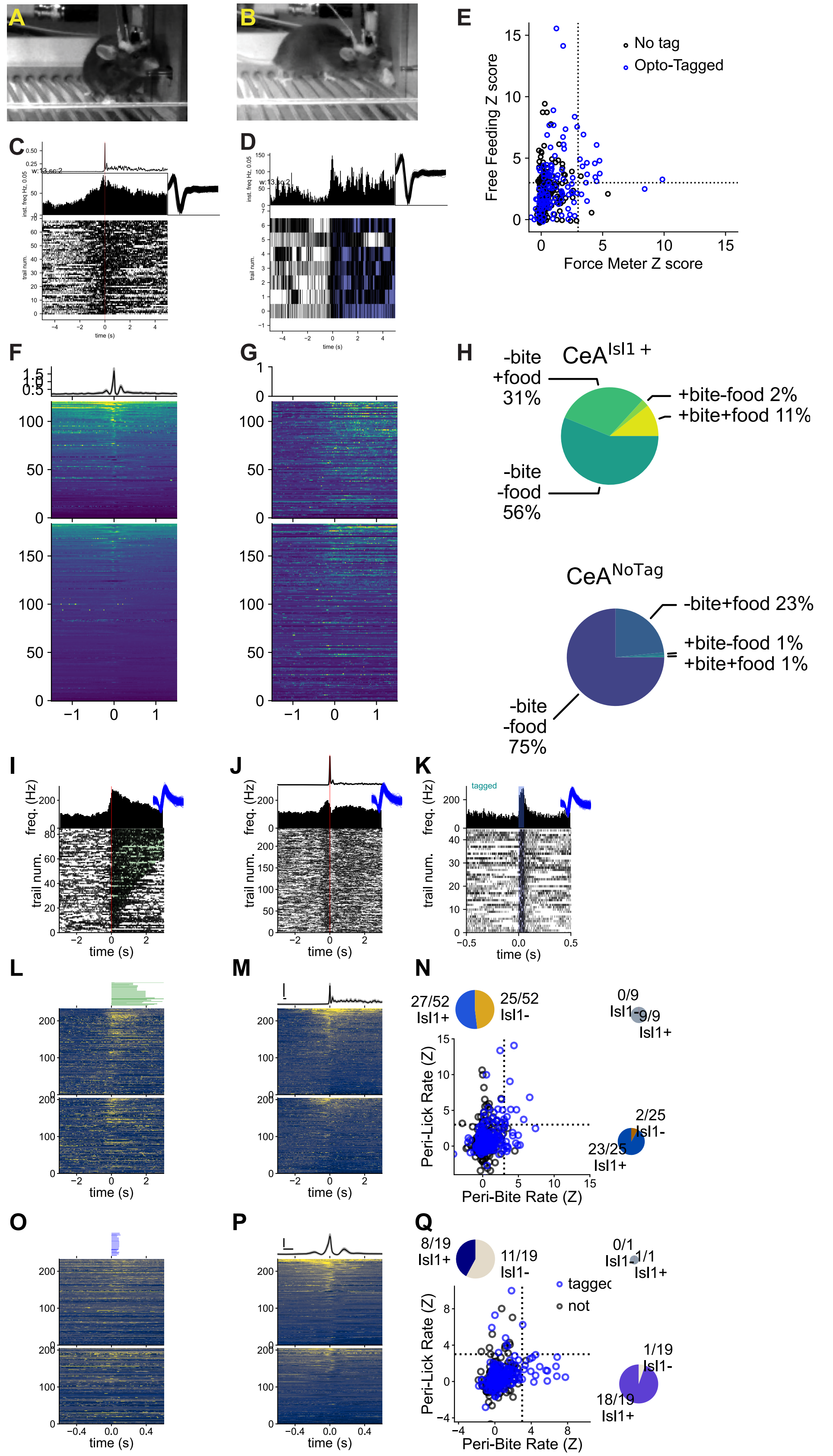

Figure S2: Opto-tagged CeA Isl1+ units are active during forceful biting, and during biting of chow.

A-B. In some recording sessions, following recording of unit activity during operant biting of force meter, chow was added to the cage and neurons were recorded during consumption of chow. Video analysis of these sessions was used to assign onset of feeding times. Here a frame from the video is shown during the first segment of the session, where the animals is biting the force meter for an infusion reward. B. Frame from video marked as time of biting food comparable to A. C. Raster showing activity of a representative opto-tagged unit during biting of the force meter. D. Raster showing activity in response to initiation of a feeding bout. Same unit as shown in (C). E. Comparison of Z

bioRxiv preprint doi: https://doi.org/10.64898/2026.08.11.744231; this version posted August 17, 2026. The copyright holder has placed this preprint (which was not certified by peer review) in the Public Domain. It is no longer restricted by copyright. Anyone can legally share, reuse, remix, or adapt this material for any purpose without crediting the original authors.

transformed firing rates during biting of the force meter (X axis) as compared to biting of chow (Y axis) for the each unit recorded with data for both behaviors. Dashed lines show an significance cut off at 3 Z units. Note that opto-tagged units are active both during consumption of food and forceful biting of the strain gauge. F. Heat map of aggregate activity of all units during biting of force meter. color scale −1 : 3 Z units. Top heat map is from opto-tagged cells, bottom heatmap is from the non-tagged cells. Trace above the heat map shows the average force applied to the force meter for all aligned bite events. G. Heat map of aggregate activity of all in responses to bouts of chow consumption. As in (F), top heat map is opto-tagged cells, bottom heatmap is non-tagged cells. No force is applied to the force meter here. Row order of the units is the same as in the heatmaps in (F). H. Pie plot showing the relative number of cells that are significantly active during biting of the force meter, and during biting of food, for both opto-tagged cells, and non-tagged cells. I,L,N. A subset CeA^{Isl1+} neurons are active during bouts of licking. I: Raster plot of representative opto-tagged unit aligned to onset of bouts of licking the water sipper. Green boxes overlayed on raster indicate the onset and offset of each licking bout. L: Heatmap of Z transformed firing rates for opto-tagged (upper) and non-tagged (lower) units, aligned to lick bout onset. Rows of heat map are ordered according to row order in panel M. Upper green boxes show the lick bouts for each record. Box height is proportional to number of bouts, box width is proportional to averaged duration of bouts. (M) Heatmaps of Z transformed firing rates for tagged (upper) and non-tagged (lower) units, aligned to 'bouts' of biting. Rows are ordered by the first principle component computed on the firing rate responses during bite bouts. N. Comparison of Z transformed firing rates during bouts of biting the force meter (X axis) as compared to bouts licking the water sipper (Y axis) for the each unit recorded with data for both behaviors. Dashed lines show the significance cut off at 3 Z units. Note that relatively fewer opto-tagged units are active during both bouts of licking and bouts of biting of the strain gauge. O-Q. Units that are significantly active during individual bites, do not show significant rate modulations about the emission of individual licks. O: Heatmap of Z transformed firing rates for opto-tagged (upper) and non-tagged (lower) units, aligned to individual licks. Rows of heat map are ordered according to row order in panel P. Upper blue boxes show the averaged lick contact time for each record. Box height is proportional to number of licks, box width is proportional to averaged duration of contact to the lick sipper. Q. Comparison of Z transformed firing rates during bites on the force meter (X axis) as compared to licks on the water sipper (Y axis) for the each unit recorded with data for both behaviors. Dashed lines show the significance cut off at 3 Z units. Note only a single unit is significantly active during both behaviors.

bioRxiv preprint doi: https://doi.org/10.64898/2026.08.11.744231; this version posted August 17, 2026. The copyright holder has placed this preprint (which was not certified by peer review) in the Public Domain. It is no longer restricted by copyright. Anyone can legally share, reuse, remix, or adapt this material for any purpose without crediting the original authors.

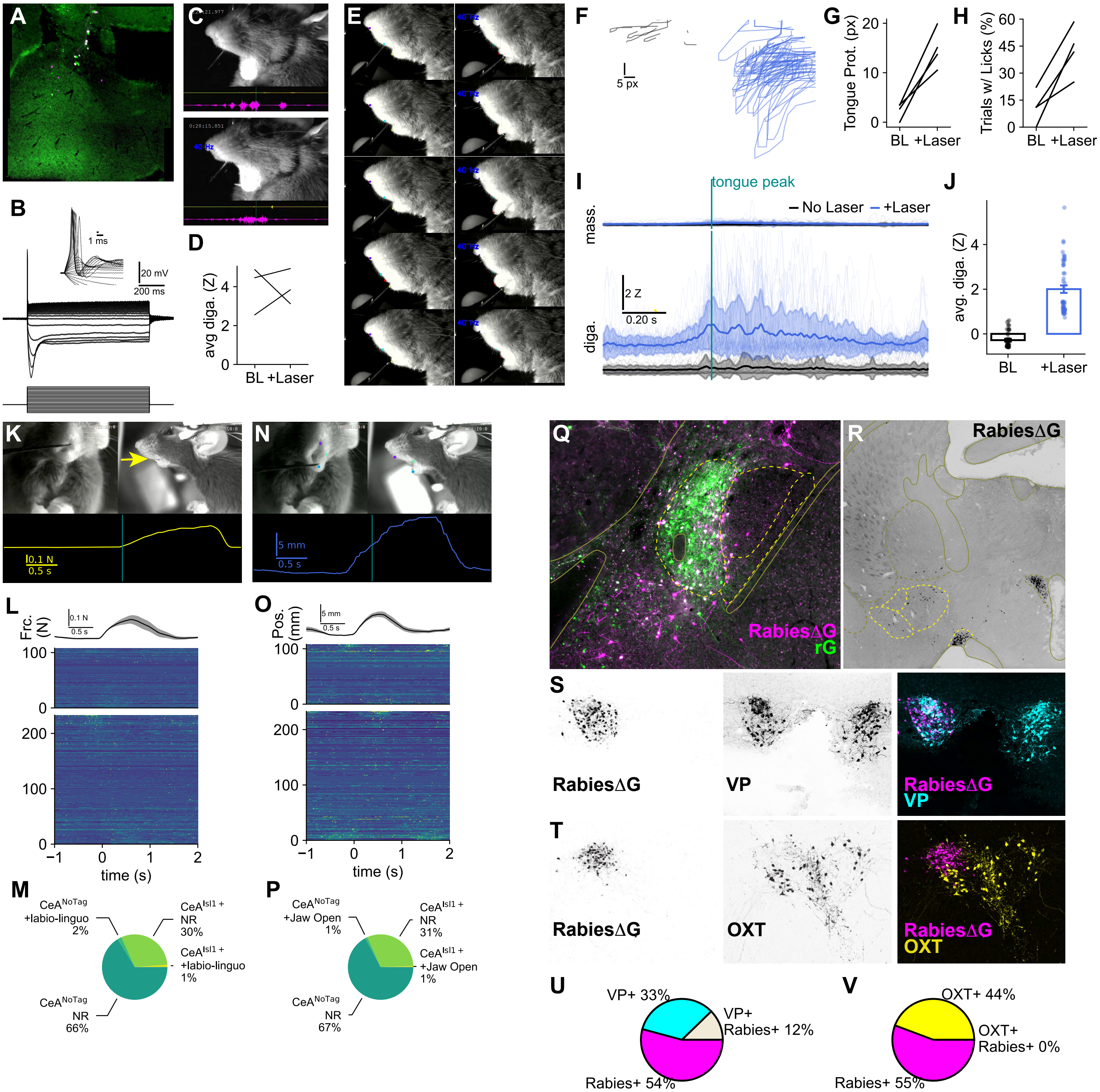

Figure S3: CeA::Isl1+ neurons do not respond to orofacial stimulation, and have a pattern of synaptic input suggesting limited direct sensory input.

A. Example micrograph of Me5 rabies starter neurons, cells expressing the rG helper (green) and rabies (magenta) appear white. These neurons are the starter cell population for mono-synaptic retrograde tracing that identified CeA^{Isl1+} cells as presynaptic. B. Example recording of family of current steps used to identify Me5 neuron by intrinsic electrophysiological properties. Note the prominent sag with hyperpolarizing steps, and the short time scale membrane resonance. C-D. Stimulation of CeA^{Isl1+} neurons does not suppress the Jaw Opening Reflex (JOR). C. Representative frame showing the JOR evoked by pressing on the hard pallet. Upper panel no stimulation, lower panel JOR evoked during 40 Hz stimulation of CeA^{Isl1+} neurons. D. Quantification of total digastric (jaw opening muscle) EMG signal elicited by pressing on hard pallet. E-I. Stimulation of CeA^{Isl1+} neurons in urethane anesthetized mouse can potentiate appetitive licking in response to brushing the upper lips. E. Two sequences of still frames showing potentiation of licking by laser stimulation during lip stimulation. Left columns of still frames shows responses to lip brushing without laser activation. Right columns shows responses to a similar manual stimulation of the upper lips which occur simultaneous to high frequency activation of CeA^{Isl1+} neurons by laser activation. Tongue position was tracked by DLC, small red dot on tongue tip shows location assignments. F. Comparison of tongue trajectories elicited by upper lip stimulation without (black)

bioRxiv preprint doi: https://doi.org/10.64898/2026.08.11.744231; this version posted August 17, 2026. The copyright holder has placed this preprint (which was not certified by peer review) in the Public Domain. It is no longer restricted by copyright. Anyone can legally share, reuse, remix, or adapt this material for any purpose without crediting the original authors.

and during (blue) laser activation of CeA^{Isl1+} neurons. Tongue protrusion distance is increased by laser activation (G) as is likelihood to elicit licking (H). I. Contraction of the digastric muscle (as measured by EMG) in response to lip touching is increased by concurrent CeA^{Isl1+} activation. Trials are aligned by the timing of the peak of tongue protrusion. Digastric EMG is significantly greater when laser stimulus is added to the lip brushing stimulation (J). K. Video visualization of experimental set up to measure single unit activity in the central amygdala during stimulation of the teeth. Example of stimulation of the incisor teeth in the labio-linguo direction. Upper frames show synchronized head-on and side view video recording. The yellow arrow on the right video frame indicates the direction of force applied to the incisors. The probe was pressed against the labio surface of the teeth. Lower yellow trace shows the force applied by manual stimulation to the incisor, upward deflection indicates the labio-linguo direction. The vertical cyan bar transecting the force trace indicates the time of the above video frames. N. Example of trial where the lower jaw is manually opened, by gently depressing the mandible. Upper frames show synchronized video record, note the purple, green, and blue dots on the video frames, these indicate the position of tracked features on each frame of video. The blue trace below the video frames plots the displacement in the position of the blue dot, from its resting position. L. Summary heat map data showing single unit responses to stimulation of incisor teeth in the labio-linguo direction. Black trace above the heat maps shows the average force applied across each recording site, gray shading indicates standard error of the mean. 341 cells in total were recorded from three mice. Four site were recorded in each mouse, for a total of 12 distinct recording locations. Of the recorded cells, 108 were classified as Opto-tagged by the SALT criteria, they are labeled as CeA::Isl1+. The remaining 233 cells are labeled as CeA::NoTag. The upper heat map shows responses of the CeA::Isl1+ cells to the tooth stimulation, the lower heat map shows the CeA::NoTag cells. The heat map spans from +3Z (yellow) to −1.5 Z (dark blue). O. Summary heat map data showing single unit responses to manual opening of the jaw. Black trace above the heatmaps shows the movement of the mandible from its rest position, averaged across the recording sites. Upper heat map shows responses of CeA::Isl1+ (opto-tagged cells), lower heat map shows responses of CeA::NoTag cells. M. Pie plots showing proportion of CeA::Isl1+ and CeA::NoTag that significantly respond to stimulation of incisors in the labio-linguo direction. P. Pie plots showing proportion of cells significantly responding to manual jaw opening. Q-V. Rabies virus mapping of inputs to CeA^{Isl1+} cells Q. Example of starter population for synaptic input mapping in the central medial amygdala. Rabies G protein is expressed by Cre dependent recombination in $Isl1^{Cre}$ mice. A small injection of the rabies G helper virus labeled the medial division of the central amygdala. Subsequent injection of ΔGrabies expressing RFP (shown in magenta), double labeled cells (white) are starter population for which synaptic inputs are mapped. 4 mice were included, all cases where similar in the extent and location of the starter injection, all injections are unilateral. R. Tiling image of the anterior portion of the central amygdala, showing the prominent inputs from the Supraoptic Nucleus (SON), and the Periventricular Hypothalamus (PVH). S-V. PVH neurons projecting to CeA^{Isl1+} neurons comprise vasopressin expressing neurons but not oxytocin expressing neurons. S. Representative immunostaining for vasopressin in PVH sections showing rabies virus retro-synaptic labeling from CeA^{Isl1+} neurons. T. Representative immunostaining for oxytocin in rabies injections cases. U. Quantification of PVH cells positive for rabies, for vasopressin, or both. V. Quantification of PVH cells positive for rabies, for oxytocin, or both.

bioRxiv preprint doi: https://doi.org/10.64898/2026.08.11.744231; this version posted August 17, 2026. The copyright holder has placed this preprint (which was not certified by peer review) in the Public Domain. It is no longer restricted by copyright. Anyone can legally share, reuse, remix, or adapt this material for any purpose without crediting the original authors.

with EMG wires in the masseter and digastric to examine the effect of CeA^{Isl1+} cell inhibition. Frame shows synchronized EMG and behavior recording, traces below still show masseter (yellow), and digastric (orange). F. Example traces of masseter and digastric EMGs, when CeA^{Isl1+} neurons are inhibited starting after initiation of pasta eating. Top trace, masseter, lower trace digastric, pull out details show the masseter burst detection (blue circles), and amplitude measurement (red enveloping line). Cyan bar above top trace indicates period during which laser light is constantly active. G. Summary data, CeA^{Isl1+} inhibition reduces the amplitude of masseter EMG bursts during feeding $t_{(4)}=5.27$, P=0.0062 H-K. Activation or inhibition of CeA^{Isl1+} neurons alters the location preference of animals in a real-time online place preference assay. H. Heatmaps showing the location preference for an animal expressing ChR2 in CeA^{Isl1+} neurons, before the activation of the laser rule (upper heat map) and again after the laser is conditionally activated (lower heat map) when the animals is present in Zone A. The laser is pulsed at a relatively low frequency, 1 Hz, that is not seen to evoke oromotor behaviors. I. Similar to H, but in an animal expressing the inhibitory opsin GtACR in CeA^{Isl1+} neurons. In the lower heat-map, the laser is continuously on while the animal is in Zone A, as compared being pulsed at 1 Hz while the animal is in Zone A in Panel H. For both of the lower heat maps in H and I, the laser is off while the animal is in Zone B. J. Summary place preference data for groups of animals expressing GtACR or ChR2. A significant interaction between activation of the laser and opsin type is observed by mixed ANOVA analysis. $F_{(1,14)}=23.67$,P=0.0002. With the laser-place rule 'online', a significant difference between preferred side is apparent between the ChR2 and GtACR groups. K. Locomotor speed was measured during the two phases of the experiment. A significant decrease in locomotor walking speed was observed in the ChR2 group.Despite this effect the average velocity between the ChR2 and GtACR groups was not different while the laser rule was online. L-N. Contingent activation of CeA^{Isl1+} neurons acts as an operant reinforcer in a nose-poke self-stimulation paradigm. L. Diagram showing paradigm of self-reinforcement design. Nose-pokes into the laser paired port activated a brief train of stimulatory laser light. Nose-pokes into the other port had no effect. M. Example event plot of poke events for one experimental subject. Experiment was conducted on consecutive days, with animals food deprived on the first day, and then the next day following reintroduction of ad lib food. N. Animals nose-poked into the laser paired port significantly more than the unpaired port. No significant difference was observed between number of pokes into the laser port when animals were food deprived as compared to ad. lib. fed. O-R. Inhibition of CeA^{Isl1+} neurons does not reduce food intake, but effects feeding proficiency. O. $Isl1^{Cre}$ mice expressing either hM4Di (inhibition, N=7) or mCherry (control, N=3) were allowed free access food available from FED3 feeders. Once a stable circadian pattern of intake was established, animals were dosed with Veh-CNO(4mg/kg)-Veh on three successive days two hours after lights out when mice began to consume most of their food. No significant differences in food intake was observed. P-R. Consumption of regular chow food intake was measured in the same animals, in a bedding free arena. Q. Weight of chow consumed was not significantly reduced by CNO treatment, but more of the food was shredded (R).

bioRxiv preprint doi: https://doi.org/10.64898/2026.08.11.744231; this version posted August 17, 2026. The copyright holder has placed this preprint (which was not certified by peer review) in the Public Domain. It is no longer restricted by copyright. Anyone can legally share, reuse, remix, or adapt this material for any purpose without crediting the original authors.

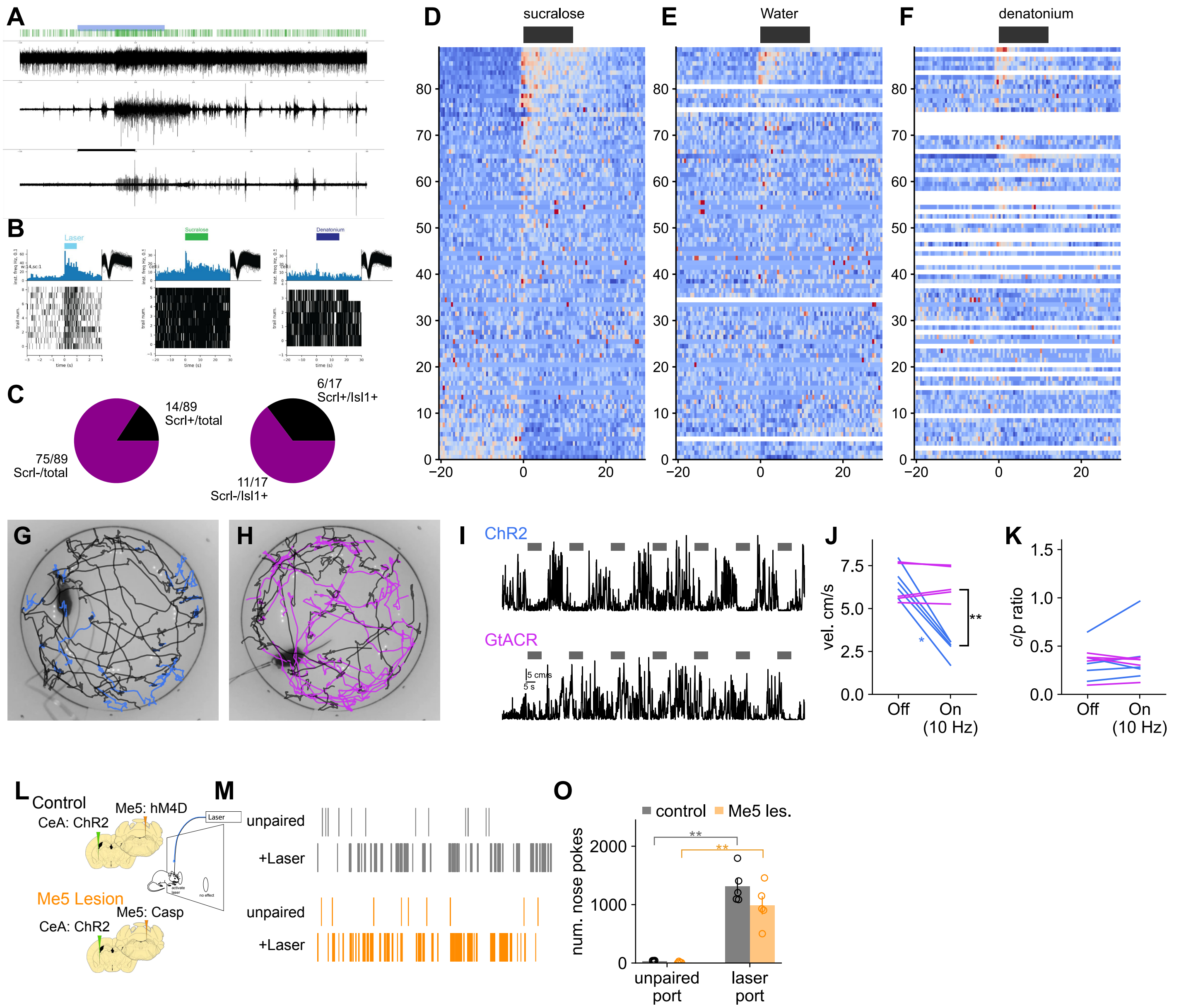

Figure S4: Central Amygdala neurons expressing Isl1 neurons are activated during consummatory motor responses evoked by sweet tastes. Activation of CeA:Isl1+ neurons halts ongoing locomotion in an open field.

A. Example trace of single unit recording of optically tagged putative CeA^{Isl1+} unit. Top trace is single unit recording, green ticks above top trace are spikes for an identified unit, the cyan bar indicates the infusion of sucralose into the oral cavity. Middle trace is digastric EMG, bottom trace is masseter EMG. The burst of EMG activity indicates the initiation of an appetitive oral response. B. Raster of responses to laser stimulation (left), sucralose (middle) and denatonium (right). Rasters to taste responses are aligned to the initiation of EMG activity observed after infusion of the tastant. C. Proportion of cells responding to sweet tastes is higher among optically tagged CeA^{Isl1+} than in the general population of amygdala neurons, fishers exact test [P=0.024]. D-F. Heatmap of all recorded neurons responses to sweet (D), water (E), and the bitter tastant, denatonium (F). G-K. Stimulation of CeA^{Isl1+} neurons reduces ongoing locomotion in an open field arena. G. Representative position tracks over 8 trials of laser stimulation in the open field. Each trail is represented by a single track, the first 8 seconds of the track, during which the laser is off are colored black, the subsequent 8 seconds of the trail, during which the laser is on, is colored in royal blue (ChR2 group). Laser activation at 10 Hz essentially dramatically reduces ongoing locomotion. H. Similar to G, but showing representative tracks for 8 trails of laser activation for the animals expressing GtACR in CeA:Isl cells. The portion of the trial track occurring during laser activation is colored in magenta (GtACR group). The laser in continuously on rather than pulsed at 10 Hz in this group. I. Upper trace: Representative velocity traces over time during 7 trails of laser activation for an animal expressing ChR2 in the CeA:Isl1 cells. Period of 10 Hz laser activation are shown by the presence of gray bar above the velocity trace, shared scale bar in lower trace. Lower trace: similar to upper, but animal is expressing GtACR in CeA:Isl1+ neurons, and laser is continuously active during periods shown by gray bar. J. Higher frequency activation (10 Hz) of CeA^{Isl1+} neurons in an open field arena significantly decreased velocity, but inhibition had no effect on walking speed $F_{(1,8)}$=177.22, P=9.7*10^-7, interaction OPSIN X LASER. Pairwise tests showed that the significant difference in walking

bioRxiv preprint doi: https://doi.org/10.64898/2026.08.11.744231; this version posted August 17, 2026. The copyright holder has placed this preprint (which was not certified by peer review) in the Public Domain. It is no longer restricted by copyright. Anyone can legally share, reuse, remix, or adapt this material for any purpose without crediting the original authors.

speed between the two group (activation and inhibition) was only observed during the laser active period ($t_{(8)}$=-7.3,P=0.0000837), during the laser off periods, the two groups walking speeds were not significantly different ($t_{(8)}$=0.29,P=0.78) K. Higher frequency CeA activation (10 Hz) or inhibition (constant illumination 3-5 mW) did not significantly shift animals localization within the open-field arena between the center and the periphery. (mixed ANOVA $F_{(1,8)}$= 1.63, P=0.237 interaction OPSIN X LASER) O-Q. CeA:Isl1+ neuron activation continues to act as an operant reinforcer in animals that cannot generate high force bites. O. The operant self-stimulation paradigm is the same as in panel L, but the viral expression and surgical approach has been extended: in addition to expression of ChR2 in CeA:Isl1+ cells, a Cre dependent caspace virus was also injected in the mesencephalic trigeminal nucleus, (Me5). Because Me5 neurons also express Isl1, they will contain Cre, and the Caspase virus will lesion these neurons. P. Example of poke events into the laser paired port and unpaired port, Similar to M, except the upper event plot (gray) is from a representative Control animal, expressing HM4:mCherry in Me5 neurons. Lower event plot (orange) is from a representative animal expressing with Me5 neurons lesioned via Caspase expression. Q. Control and Me5 lesioned animals nose-poked into the laser paired port significantly more than the unpaired port, mixed ANOVA: pokes ~ port * lesion + Error(subject/port), port: $F_{(1,8)}$=116.09. No significant interaction was observed between the lesion and port: $F_{(1,8)}$=2.14,P=0.182. Post hoc tests confirmed that both animals in the control ($t_{(4)}$=-9.27,P=0.00075) and intact ($t_{(4)}$=-6.196,P=0.0034) groups poked more into the laser paired port than the unpaired port. Further, post-hoc tests comparing the pokes into the laser paired port between the lesion and control groups did not reveal a significant difference ($t_{(8)}$=1.601,P=0.1479).

bioRxiv preprint doi: https://doi.org/10.64898/2026.08.11.744231; this version posted August 17, 2026. The copyright holder has placed this preprint (which was not certified by peer review) in the Public Domain. It is no longer restricted by copyright. Anyone can legally share, reuse, remix, or adapt this material for any purpose without crediting the original authors.

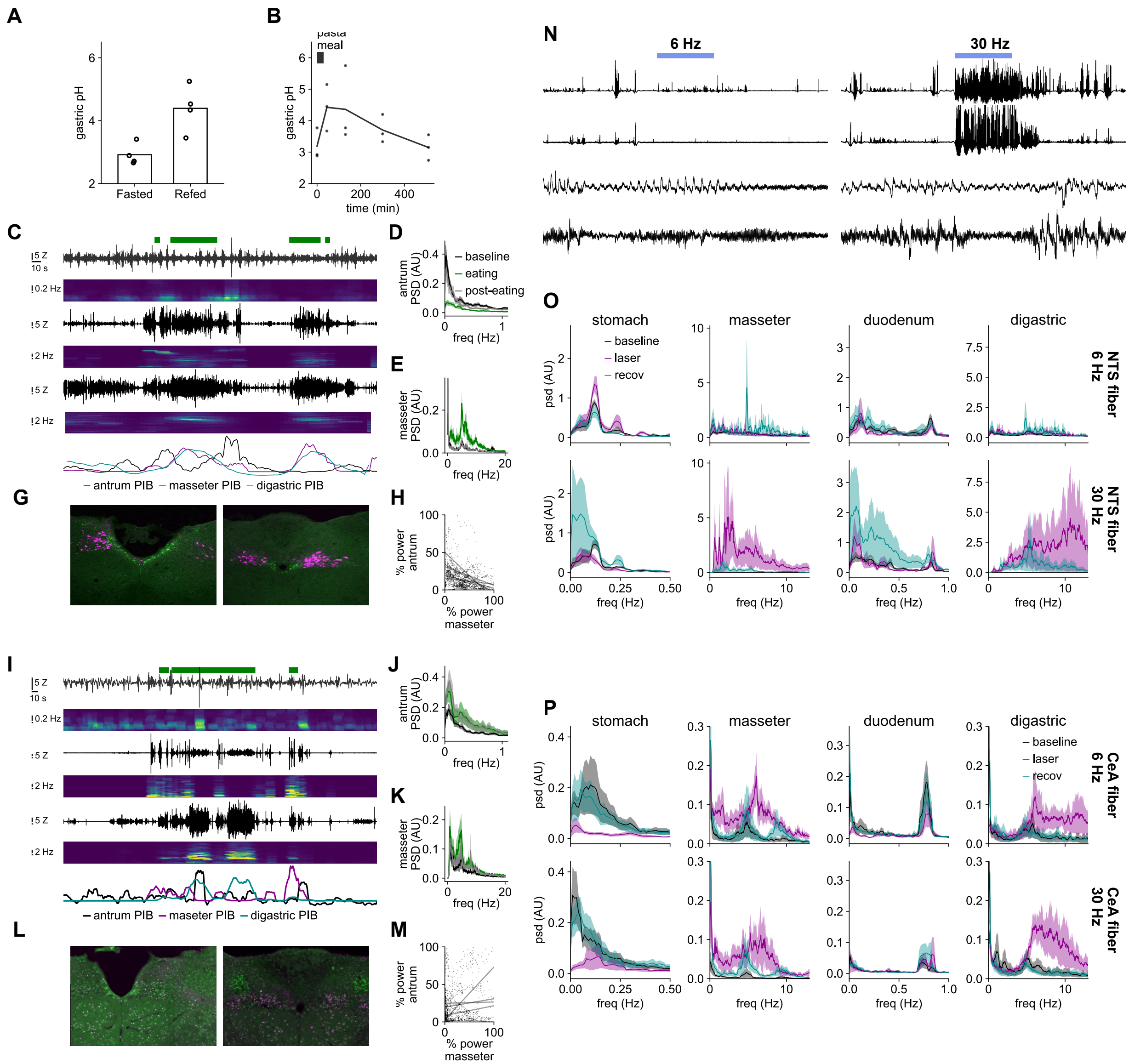

Figure S5: Suppression of the gastric antral rhythm observed during CeA^{Isl1+} stimulation is also observed during consumption of food, this phenomenon depends on the integrity of the vagus nerve. A-B. Gastric pH is increased by the consumption of food. A. Gastric pH was measured by terminal collection of gastric contents either after a 12 hour fast, or 2 hours after last access to food. Faster gastric pH is lower than the re-fed condition. B. Measurement of time course of increase in gastric pH following a pasta meal. Chronic gastric fistula animals (N=3). C. Representative EMG recordings of gastric and jaw signals before, and during, consumption of standard chow. Traces and spectrograms are in pairs: upper is gastric antrum, middle is masseter, lower is digastric. Note the frequency range for the gastric antrum spectrogram is much lower than for the masseter and the digastric. Green bars above top trace indicate the period of time animal is consuming chow. Line plots at the bottom show the total power in the relevant frequency band for the gastric antrum and for the maseter. D. Consumption of food suppresses the electrical rhythm of the gastric antrum, the amplitude of the power spectrum between 0 and 0.3 Hz is significantly reduced (t[4]=7.06, P=0.0021) during food consumption as compared to the baseline period immediately preceding consumption. E. Power spectral changes in masseter activity during food consumption. F. A significant negative correlation between the amplitude of the power spectrum of the antral rhythm and the amplitude of the chewing rhythm in the masseter is observed in 5/5 intact animals. G. Retrograde transport of cholera toxin subunit b (ctb) following injection in the stomach in an intact animal. I-M. Gastric motility is not suppressed during chow consumption in animals receiving a bilateral subdiaphragmatic vagotomy. I. Example traces as in panel B. J. Power spectrum as in panel D, note that while food consumption has an effect in vagotomized animals, ($F_{(2,6)}$=6.9, P=0.028), post hoc tests suggest an increase in gastric antral activity immediately following consumption ($t_{(3)}$=-4.3, P = 0.023). K. Power spectrum as in C, in vagotomized animals. M. None of vagotomized animals had a significant negative correlation between masseter and antral activity. L. Following ctb injection in the stomach of vagotomized animals, very little labeling is observed in the dorsal vagal complex. N. Representative traces EMG activity of the (from top to bottom): digastric, masseter, gastric antrum, and duodenum in response to stimulation of CeA^{Isl1+} terminal fibers in the NTS at 6 Hz (left) and 30 Hz (right). Note the potentiation of the gastric rhythm in response to 6 Hz, as compared to the inhibition observed in response to 30 Hz stimulation. O. Average power spectral density estimates from mice with fiber position in above the NTS for stimulation of CeA^{Isl1+}

bioRxiv preprint doi: https://doi.org/10.64898/2026.08.11.744231; this version posted August 17, 2026. The copyright holder has placed this preprint (which was not certified by peer review) in the Public Domain. It is no longer restricted by copyright. Anyone can legally share, reuse, remix, or adapt this material for any purpose without crediting the original authors.

terminals projecting to this region. Upper row, are changes in response to laser stimulation at 6 Hz, lower row are changes in response to 30 Hz. P. Average power spectral density estimates from mice with fiber positioned above the central amygdala for stimulation CeA^{Isl1+} cells independent of projection target, plots are otherwise comparable to those in panel O, save absolute amplitudes are different.
